# The H3ABioNet helpdesk: an online bioinformatics resource, enhancing Africa’s capacity for genomics research

**DOI:** 10.1186/s12859-019-3322-3

**Published:** 2019-12-30

**Authors:** Judit Kumuthini, Lyndon Zass, Sumir Panji, Samson P. Salifu, Jonathan K. Kayondo, Victoria Nembaware, Mamana Mbiyavanga, Ajayi Olabode, Ali Kishk, Gordon Wells, Nicola J. Mulder, as members of the Sustainability and Outreach Work Package of the H3ABioNet Consortium

**Affiliations:** 1 Centre for Proteomic and Genomic Research, Cape Town, Western Cape South Africa; 2 Computational Biology Division, IDM, Faculty of Health Sciences, University of Cape Town, Cape Town, Western Cape South Africa; 3 Kumasi Centre for Collaborative Research in Tropical Medicine, Kwame Nkrumah University of Science and Technology, Kumasi, Ghana; 4 Uganda Virus Research Institute, Entebbe, Uganda; 5 Center for Informatics Science, Nile University, 6th October City, Egypt

**Keywords:** Bioinformatics support, Genomics support, H3Africa, H3ABioNet, Helpdesk

## Abstract

**Background:**

Currently, formal mechanisms for bioinformatics support are limited. The H3Africa Bioinformatics Network has implemented a public and freely available Helpdesk (HD), which provides generic bioinformatics support to researchers through an online ticketing platform. The following article reports on the H3ABioNet HD (H3A-HD)‘s development, outlining its design, management, usage and evaluation framework, as well as the lessons learned through implementation.

**Results:**

The H3A-HD evaluated using automatically generated usage logs, user feedback and qualitative ticket evaluation. Evaluation revealed that communication methods, ticketing strategies and the technical platforms used are some of the primary factors which may influence the effectivity of HD.

**Conclusion:**

To continuously improve the H3A-HD services, the resource should be regularly monitored and evaluated. The H3A-HD design, implementation and evaluation framework could be easily adapted for use by interested stakeholders within the Bioinformatics community and beyond.

## Background

In the last decade, the ability to perform bioinformatics analyses has become crucial to biomedical science [1]. Due to collaborative efforts and increased funding across Africa, the continent has steadily developed its bioinformatics capacity over recent years [2, 3]. H3ABioNet (H3Africa Bioinformatics Network, www.h3abionet.org) is a pan-African bioinformatics network which supports such development [4, 5]. The network has successfully built capacity through various strategies, including, upgrading computing infrastructure in Africa; developing and customizing software tools to facilitate data management and manipulation; initiating collaborative multidisciplinary research partnerships, and providing blended bioinformatics learning for researchers across the continent [4–6].

Generic bioinformatics support is typically achieved through research collaboration, consultancy, and, most frequently, informal online communications [7, 8]. To our knowledge, few formal or professional mechanisms of generic bioinformatics support currently exist. Helpdesks (HDs) are commonly employed as core support resources, both academically and commercially, and function as important channels which aid the communication of complex concepts [9]. However, few bioinformatics HDs have been established, and these are often resource-, community- or region-specific [10, 11].

Due to the lack of established bioinformatics support resources available to African researchers, H3ABioNet established an online bioinformatics HD (henceforth referred to as the H3A-HD) to aid bioinformatics capacity development. To our knowledge, the H3A-HD is one of the first public and freely available, generic bioinformatics HDs, systematically implemented to provide rapid bioinformatics support across Africa and the rest of the world. The following report introduces the H3ABioNet bioinformatics HD, and outlining its function and use, and providing suggestions for future HD implementations.

### The H3ABioNet helpdesk (H3A-HD)

The H3A-HD provides bioinformatics and H3ABioNet-specific project support through technical experts (or H3A-HD representatives), with backgrounds in various bioinformatics sub-disciplines [3]. Representatives are H3ABioNet members maintained on a voluntary basis, ranging from mid- to late-career scientists, and selected based on their qualifications and research expertise. Based on the overlap between expertise and query categories, an H3A-HD administrator prioritizes and categorizes queries as simple, intermediate or advanced, before assigning them to the HD representative of the relevant query category. A given representative handles a single query category; and if an assigned query remains unresolved, the query is reassigned as necessary and the ticket history is shared across HD representatives.

A technical platform for the H3A-HD was selected on the basis of being cost free, easy to maintain and use, as well as being customizable to the existing H3ABioNet website. The tool should also allow an unlimited number of ticket categories, with an unlimited number of representatives. Therefore, an open-source tool built using Django, django-helpdesk, was selected for the front- and backend of the H3A-HD. Designed for small businesses to receive, manage and respond to requests for help from external users, or other people within your institute. Representatives can view the ticket dashboard containing assigned and unassigned tickets, as well as the basic status of the helpdesk. They are able to search through all tickets, save their searches for future use, and follow up or respond to tickets.

The H3A-HD accepts queries, submitted as tickets, through an online interface at https://helpdesk.h3abionet.org/. Users are encouraged to create logins to aid H3A-HD maintenance. An administrator assigns tickets to representatives, who resolve and close tickets. The H3A-HD supports a wide range of query categories. These categories are broadly arranged into 3 overall category types; Analysis – categories pertaining to the analysis of genetic data; H3ABioNet Tools and Services – dedicated to H3ABioNet associated projects and outputs; and Additional Support – geared to general technical support. The H3A-HD thus differs from other support platforms due to the type and level of support provided. Users can also browse and access additional resources which complement the HD services, including standard operating procedures (SOPs), workflows and pipelines for Analysis categories, and support guidelines and material for H3ABioNet Tools and Services, and Additional Support categories (Table 1).

**Table 1:** Query Categories and Standard Operating Protocols supported by the Help Desk

Overall Category Type	Query Category	Associated Workflows
Analysis	Analysis – NGS	Human Variant Calling Workflow
	Analysis – GWAS	Variant Discovery and Prioritisation
	H3Africa Genotyping Array	GWAS Pipeline
	Microbiome Analysis	RNA-seq Workflow
	Chip Imputation	16S rRNA Diversity Analysis Workflow
	Variant Calling Analysis	Imputation Pipeline
	Machine Learning	
	Biostatistics	
H3ABioNet Tools & Services	Standard CRF	Associated information packets, guidelines, and tools.
	Minimum Data Standards	
	General Queries	
	Human Mutation Analysis Platform (HUMA)	
	Job Management System (JMS)	
	Node Accreditation	
	NetCapDB	
	Website/Mailing List	
Additional Support	REDCap	Linux: Getting Started
	Data Management	Linux: Configuring and securing your server
	Software Programming	Install, configure and use Globus
	Software License Request	Online to transfer large datasets
	System Administration	Setup and configure a basic HPC cluster

## Results

The H3A-HD is regularly monitored and evaluated using built-in Joomla functionality, qualitative analysis and user feedback.

### Usage and query resolution time

**Fig. 1:**
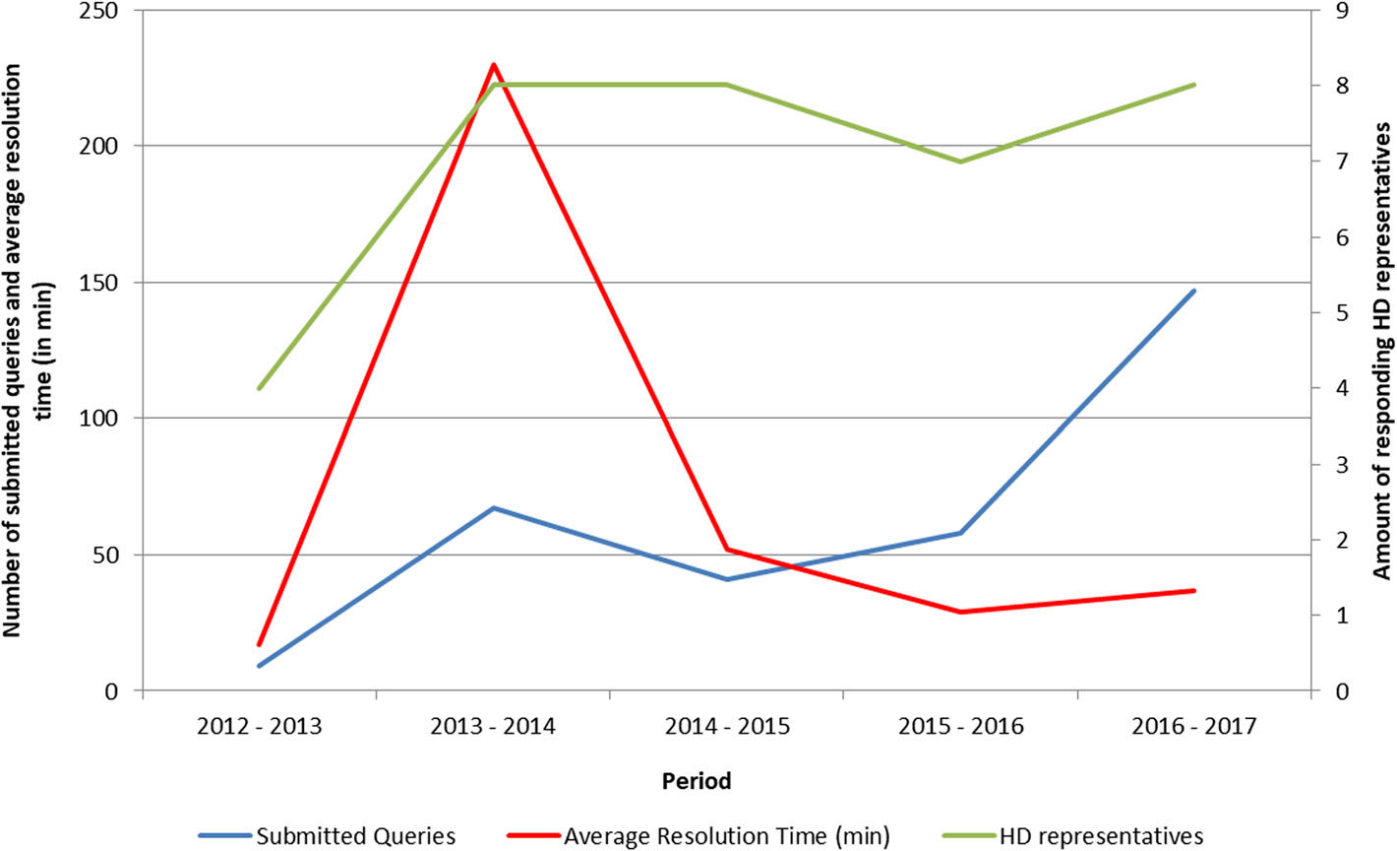
Distribution of submitted queries, H3A-HD representatives and average resolution time from 1 August 2012 to 1 August 2017

Based on the automatically generated logs, 320 queries were submitted to the H3A-HD between 1 August 2012 and 1 August 2017. Figure 1 illustrates the query volumes, average resolution times and the number of responding HD representatives, during this period. The left axis illustrates the distribution of query volume and average resolution time (in minutes), and the right axis illustrates number of responding H3A-HD representatives, over time.

There was an overall increase in H3A-HD usage over time, with peak usage between 2016 and 2017. Notably, despite increased query volume and no change in the amount of responding HD representatives over time, there was an overall decrease in query resolution time. This indicates the HD representatives’ improved capacity and query knowledge over time.

### Feedback

**Fig. 2:**
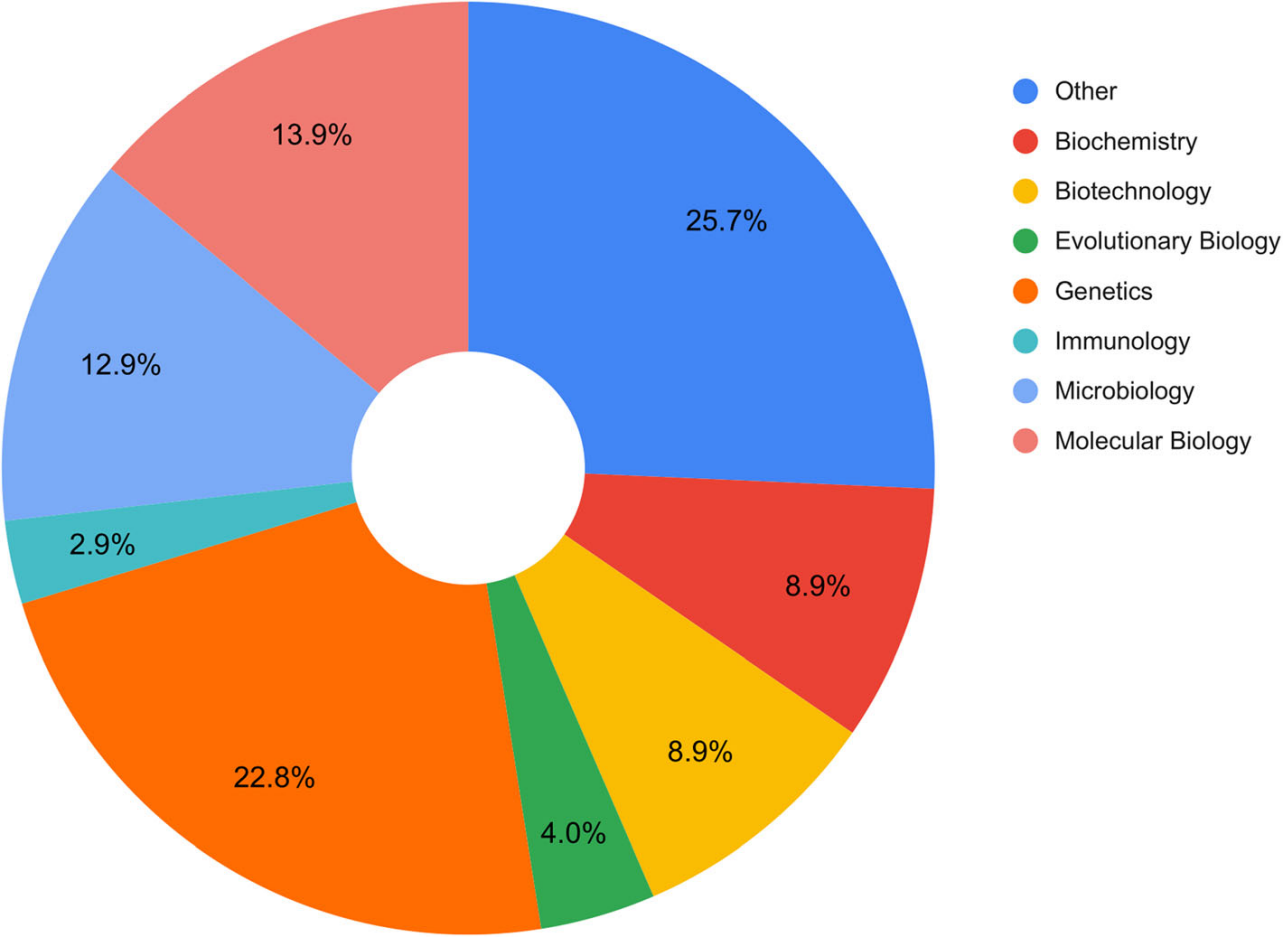
Distribution of survey respondents’ research fields

Feedback from previous H3A-HD users and potential network users was determined using an online survey, distributed to 244 participants. Of these participants, 128 (53%) responded; the majority (75%) had not previously used the H3A-HD, stating ‘not yet required’ (53%) and ‘lack of awareness’ (24%) as the primary reasons. Others (19%) expressed a need for category expansion, requesting cytogenetics, metagenomics, deep learning etc. The remaining respondents (25%) had previously used the H3A-HD. The majority of respondents had mixed research backgrounds (26%), followed closely by genetics (23%) (Fig. 2).

**Fig. 3:**
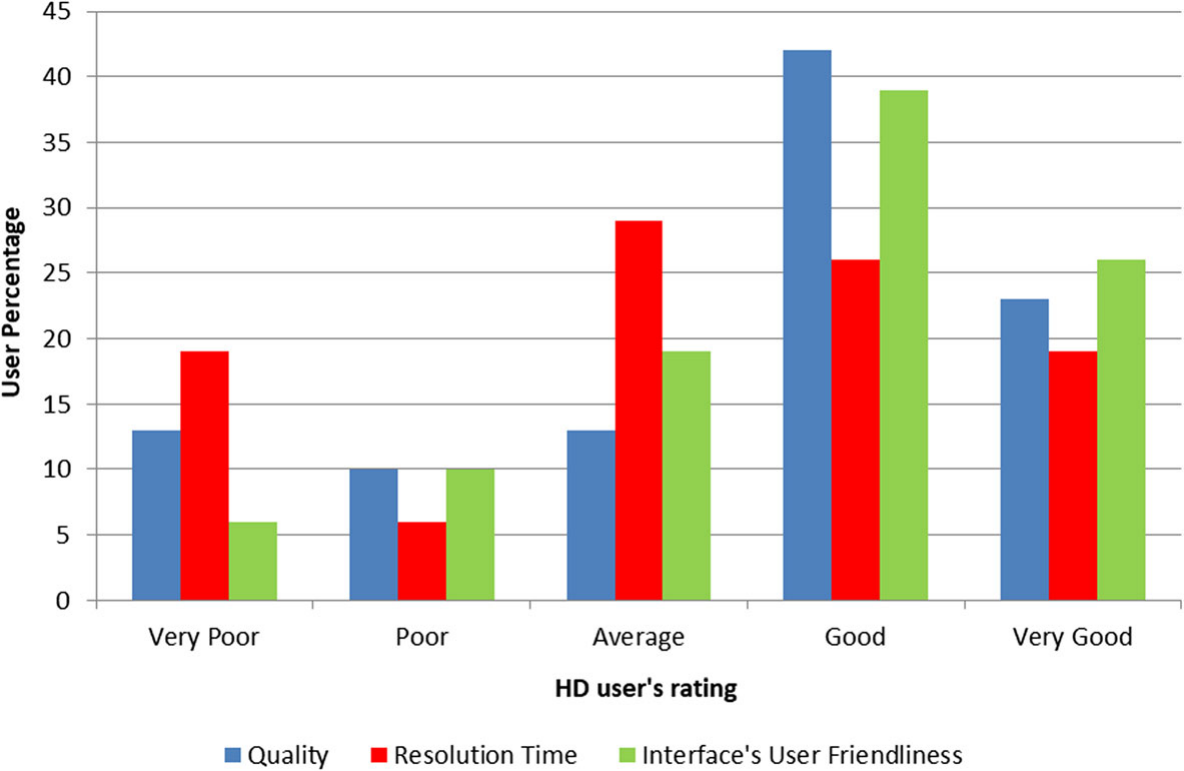
H3A-HD users’ evaluation of HD’s quality, resolution time and user friendliness

As illustrated in Fig. 3, H3A-HD user satisfaction was measured on a standard 1–5 scale (very poor, poor, average, good, very good), assessing the quality of support received, resolution time and user friendliness of the H3A-HD interface. The majority of H3A-HD users rated quality of support (77%), query resolution time (74%), and user friendliness of the H3A-HD interface (84%), average and above.

**Fig. 4:**
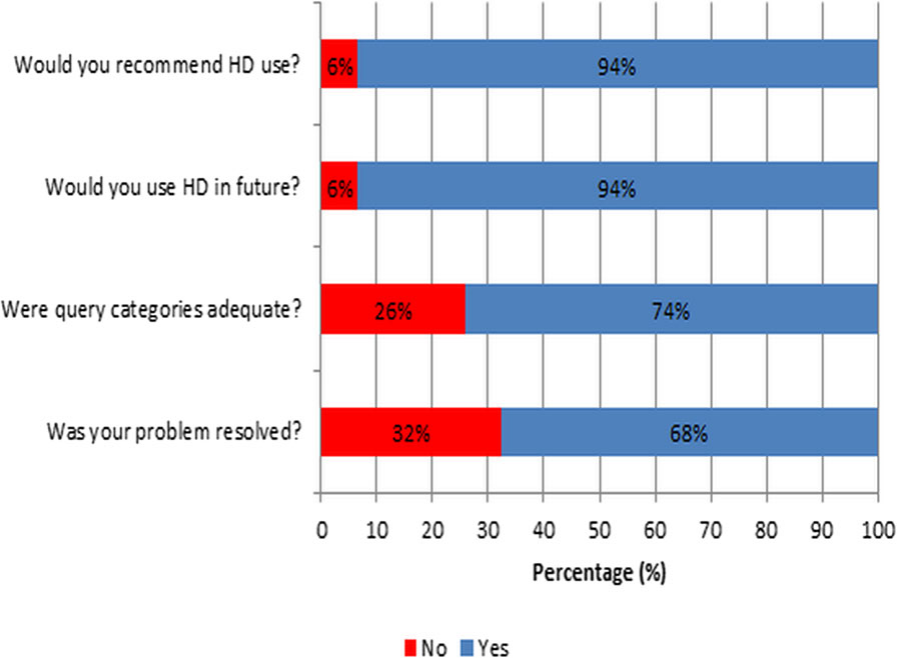
H3A-HD user satisfaction with respect to various evaluation criteria

Figure 4 summarizes H3A-HD users’ answers to a series of closed questions. Responses were generally positive and only 6% of previous users responded that they wouldn’t use or recommend the use of the H3A-HD in the future. In addition, only 30% of HD users’ queries were not resolved. These users were generally preferred more interactive communication, as they did not know who to follow up with regarding delayed response.

### Qualitative analysis

To identify recurrent themes and concerns, queries were qualitatively analysed on a ticket-by-ticket basis. Recurrent queries can be divided into two broad categories, H3ABioNet-specific queries (e.g. node accreditation; NetCapDB; software license and data requests), and queries which deal with data analysis applications (e.g. workflow, scripting and software support).

H3ABioNet-specific queries tend to accumulate with the introduction of new activities and (or) tools in the network. This is exemplified in the network’s introduction of the node accreditation project; a project which consists of exercises designed to assess the capacity of H3ABioNet nodes to properly process and analyse datasets typically produced by H3Africa. Network members typically use the Helpdesk to submit their intentions for node assessment and to obtain scripting or tool use support when conducting their assessments. Likewise, during H3ABioNet’s infancy, several queries involved the setup and installation of servers, and similar trends followed the introduction of NetCapDB, a network reporting system, and various software courses.

Project-specific queries are typically received during the planning or data analysis of a given user’s research project. Queries generated during project preparation include queries involving computing requirements, software inquiries, comparisons and recommendations. Software recommendations are generally within the scope of GWAS, WES and 16S data analysis. Queries received during data analysis involve the preparation of appropriate computing environments for data analysis, as well as scripting, particularly in relation to specific tools (e.g. FASTQ, Trimmomatic and BWA). Scripting languages may vary by query and queries also extend to the identification and resolution of errors during analysis.

## Discussion

Due to several concerted initiatives, bioinformatics capacity in Africa is on the rise [12]. However, the demand still outweighs the supply, and researchers may often be left isolated, with little support, if their associated institutes are not equipped with dedicated support units [13]. The current report has introduced the H3ABioNet bioinformatics HD, a platform which can be employed to overcome such isolation. Although, the H3A-HD primarily serves African scientists, the service is available to all individuals seeking bioinformatics support. The H3A-HD specifically serves the needs of African researchers because these concerns are not appropriately addressed by other support platforms. In addition, the HD representatives are uniquely aware and equipped to provide support for the bioinformatics challenges facing African researchers. Systematic integration of the H3A-HD into existing H3ABioNet activities and training events is essential to raising its awareness and to provide the learner community support following a training events [14].

Several lessons have been learned based on the initial implementation and evaluation of the H3A-HD which may be useful for the development and initiation of future HD iterations. Firstly, interactive communication methods are central to a HD’s functionality and effectiveness. Ineffective communication mechanisms, query disputes and miscommunication are often the cause of unresolved queries. Effective and interactive communication can be achieved through increased email correspondence or live communication mechanisms such as video calling systems [15]. Secondly, ticketing strategies should be carefully developed prior to HD implementation, as the information acquired during ticket creation is essential to the maintenance and monitoring of an HD. The ticketing strategies employed may vary between HDs, and some HDs may employ more than one ticketing strategy. HDs commonly create tickets automatically via email correspondence, using Jira-based tracking [11] or case-based reasoning techniques [16]. Thirdly, the system used to implement the HD should be carefully evaluated based on the HD’s aim. The H3A-HD makes use of a closed system because it may support research-specific queries, which may require privacy. Additionally, implementing the H3A-HD using a pre-existing and open source platform proved advantageous in terms of developmental work, however, using a supported platform may prove beneficial in the future, as relying on an unfunded, and open-source platform has the potential to compromise technical support and platform upgrading [17].

A number of key factors should be considered for future HD implementations. An HD should develop clear guidelines which outline the boundaries between query support and collaborative work. In the current report, assessment of user satisfaction is limited to a once-off, retrospective survey; however, best practice requires obtaining user feedback on a query-by-query basis. Such evaluation is crucial, and should be implemented in future H3A-HD iterations, as it provides real time evaluation, and may inform immediate alterations to the H3A-HD. A crucial limitation of the current evaluation is the omission of HD representatives. Reviews of an HD system should aim to assess the platform, the representatives, users and the management team to comprehensively identify key areas of success and improvement. Although a number of HDs exist within the bioinformatics and biological sciences community, associated publications are scarce, and there is a gap in helpdesk literature to compare to [10, 11, 16]. We hope to encourage others to publish their implementations, in order to build a knowledgebase for future implementations to build on.

To effectively sustain the H3A-HD, linkages with organizational bodies should be developed to sustain both financial and management support. Funding is an important component of the implementation, sustainability and success of an HD. Funded HDs generally have higher satisfaction rates than observed in the current report, however, the extent to which funding influences the quality of support provided by the H3A-HD and cannot be fully elucidated without evaluating the HD volunteers. Sustainability may require the production of a more comprehensive H3A-HD resource through an increased number of HD representatives and (or) expansion of supported query categories. Additionally, support should be continuously updated and maintained by allowing common queries to guide SOP updates.

## Conclusion

H3ABioNet effectively launched one of the first free bioinformatics HDs in Africa, a flagship resource providing support to African researchers and beyond. Notably, communication mechanisms, ticketing strategies and usage frameworks are crucial to running a successful HD, and should be considered when developing or upgrading an HD. Because HDs are becoming crucial components of scientific research, more HDs need to make their design, management, usage and evaluation frameworks publically available, either online or through publication, to guide the development and implementation of future HDs. This will ensure that the gaps and strengths of existing HDs are easily identifiable and addressed, if necessary.

The H3A-HD has a proven track record of effective query management and user satisfaction. As a crucial component of H3ABioNet’s support mechanism, the H3A-HD envisages becoming an important contributor to the effective increase of bioinformatics capacity in Africa. We encourage other HDs to make their development processes publicly available, as it may aid similar efforts in the effective development of their HDs.

## Methods

To monitor progress and deliver improvement strategies, H3ABioNet frequently audits the H3A-HD using automatically generated logs accessible from the Joomla platform. The logs include query volume and resolution time. For this report, logs from the Joomla platform were analysed from August 2012 to August 2017.

A retrospective user satisfaction survey was created to assess user satisfaction with regards to support quality, resolution time and user friendliness, as well as future use and recommendation, from the users’ perspective (see Additional file 1). The online and anonymous feedback survey was distributed to 244 participants to assess levels of user satisfaction. Data from the survey was collected and managed using REDCap (Research Electronic Data Capture) version 7.5.0, electronic data capture tools, hosted at The Centre for Proteomic and Genomic Research (CPGR).

Qualitative evaluation was also conducted based on the submitted queries to identify relevant themes with regards to the submitted queries and identify the types of applications in which African scientists require assistance.

## Supplementary information

**Additional file 1.** Usesr Satisfaction Survey

## Data Availability

The datasets used and/or analysed during the current study are available from the corresponding author on reasonable request.

## Abbreviations

H3ABioNet: H3Africa Bioinformatics Network
H3Africa: Human Heredity and Health of Africa Consortium
H3A-HD: H3ABioNet Helpdesk
HD: Helpdesk
JMS: Job Management System
SOP: Standard Operating Procedure
