# Supplementary material for: The H3ABioNet helpdesk: an online bioinformatics resource, enhancing Africa’s capacity for genomics research
Source: BMC Bioinformatics. 2019 Dec 30;20:741. doi: 10.1186/s12859-019-3322-3 (PMC6937968; doi:10.1186/s12859-019-3322-3)
Supplement: Supplementary file 1 — Additional file 1. Usesr Satisfaction Survey [file 12859_2019_3322_MOESM1_ESM.pdf]

H3ABioNet Bioinformatics Help Desk Survey

Welcome to the H3ABioNet Help Desk user satisfaction survey!

Thank you for taking part in this important survey to measure user satisfaction for the H3ABioNet Bioinformatics Help Desk (http://www.h3abionet.org/support).

The H3ABioNet Bioinformatics Help Desk provides access to experts from a variety of domains to help answer any bioinformatics-related questions and provide support to various H3Africa and non-H3Africa projects that might be struggling with the analysis and planning of their experiments.

The following survey aims to evaluate the service provided by the Help Desk and identify features to be improved and should only take a few minutes. Please be assured that all responses will be annonymized.

By continuing with the survey you give your consent for the use of the annonymized data to be analysed and published.

General Information

Name and Surname ________________________________

Country Based In

◯ Algeria
◯ Angola
◯ Benin
◯ Botswana
◯ Burkina Faso
◯ Burundi
◯ Cabo Verde
◯ Cameroon
◯ Central African Republic (CAR)
◯ Chad
◯ Comoros
◯ Democratic Republic of the Congo
◯ Republic of the Congo
◯ Cote d'Ivoire
◯ Djibouti
◯ Egypt
◯ Equatorial Guinea
◯ Eritrea
◯ Ethiopia
◯ Gabon
◯ Gambia
◯ Ghana
◯ Guinea
◯ Guinea-Bissau
◯ Kenya
◯ Lesotho
◯ Liberia
◯ Libya
◯ Madagascar
◯ Malawi
◯ Mali
◯ Mauritania
◯ Mauritius
◯ Morocco
◯ Mozambique
◯ Namibia
◯ Niger
◯ Nigeria
◯ Rwanda
◯ Sao Tome and Principe
◯ Senegal
◯ Seychelles
◯ Sierra Leone
◯ Somalia
◯ South Africa
◯ South Sudan
◯ Sudan
◯ Swaziland
◯ Tanzania
◯ Togo
◯ Tunisia
◯ Uganda
◯ Zambia
◯ Zimbabwe
◯ International

Please specify which "international" country: ______________________________

Nationality

- ◯ Algeria
- ◯ Angola
- ◯ Benin
- ◯ Botswana
- ◯ Burkina Faso
- ◯ Burundi
- ◯ Cabo Verde
- ◯ Cameroon
- ◯ Central African Republic (CAR)
- ◯ Chad
- ◯ Comoros
- ◯ Democratic Republic of the Congo
- ◯ Republic of the Congo
- ◯ Cote d'Ivoire
- ◯ Djibouti
- ◯ Egypt
- ◯ Equatorial Guinea
- ◯ Eritrea
- ◯ Ethiopia
- ◯ Gabon
- ◯ Gambia
- ◯ Ghana
- ◯ Guinea
- ◯ Guinea-Bissau
- ◯ Kenya
- ◯ Lesotho
- ◯ Liberia
- ◯ Libya
- ◯ Madagascar
- ◯ Malawi
- ◯ Mali
- ◯ Mauritania
- ◯ Mauritius
- ◯ Morocco
- ◯ Mozambique
- ◯ Namibia
- ◯ Niger
- ◯ Nigeria
- ◯ Rwanda
- ◯ Sao Tome and Principe
- ◯ Senegal
- ◯ Seychelles
- ◯ Sierra Leone
- ◯ Somalia
- ◯ South Africa
- ◯ South Sudan
- ◯ Sudan
- ◯ Swaziland
- ◯ Tanzania
- ◯ Togo
- ◯ Tunisia
- ◯ Uganda
- ◯ Zambia
- ◯ Zimbabwe
- ◯ International

Please specify which "international" country: ______________________

Institution ______________________
(In full; no abbreviations)

Field of Research	◯ Anatomy<br>◯ Astrobiology<br>◯ Biochemistry<br>◯ Biogeography<br>◯ Biological engineering<br>◯ Biophysics<br>◯ Behavioral neuroscience<br>◯ Biotechnology<br>◯ Botany<br>◯ Conservation biology<br>◯ Developmental biology<br>◯ Ecology<br>◯ Ethnobiology<br>◯ Ethology<br>◯ Evolutionary biology<br>◯ Genetics<br>◯ Gerontology<br>◯ Immunology<br>◯ Marine biology<br>◯ Microbiology<br>◯ Molecular biology<br>◯ Neuroscience<br>◯ Paleontology<br>◯ Parasitology<br>◯ Physiology<br>◯ Soil biology<br>◯ Sociobiology<br>◯ Systematics<br>◯ Toxicology<br>◯ Zoology<br>◯ Other
Have you used the Help Desk?	◯ Yes<br>◯ No
Please provide a reason for this:	◯ Never heard of it<br>◯ Not appropriate for my needs<br>◯ The interface is not user friendly<br>◯ Haven't required it thus far
Which field(s) do you require in the application of bioinformatics in human genetics?	________________________________
Query Category	☐ Analysis - Genotyping Arrays<br>☐ Analysis - NGS Data<br>☐ Analysis - Other<br>☐ Biostatistics<br>☐ Data Management<br>☐ General Project Administration<br>☐ JMS<br>☐ NetCapDB<br>☐ Netmap Installation<br>☐ Other<br>☐ Software Development/Programming<br>☐ Software License Request<br>☐ Technical/System Administration<br>☐ Website/Mailing List

Help Desk Evaluation (1)

Assess the quality of your experience using the H3ABioNet Help Desk by rating the following questions; 1 being poor and 5 being excellent:

1. Ability and quality of the support provided	◯ 1 ◯ 2 ◯ 3 ◯ 4 ◯ 5
2. Turnaround time	◯ 1 ◯ 2 ◯ 3 ◯ 4 ◯ 5
How long did processing your query take?	◯ 1 week ◯ 2 - 4 weeks ◯ 2 - 5 months ◯ over 5 months
3. User friendliness of the H3ABioNet Help Desk	◯ 1 ◯ 2 ◯ 3 ◯ 4 ◯ 5<br>((logging of tickets; query categories etc.))

Help Desk Evaluation (2)

Assess the quality of your experience using the H3ABioNet Help Desk by answering Yes/No to the following questions:

1. Was your problem resolved by consulting the Help Desk?	◯ Yes ◯ No
2. Were the query categories adequate for your needs?	◯ Yes ◯ No
3. Would you use the H3ABioNet Help Desk for future purposes?	◯ Yes ◯ No
4. Would you recommend the H3ABioNet Help Desk to others?	◯ Yes ◯ No

Additional Comments

Feel free to provide any additional commentary or recommendations to improve the services provided by the H3ABioNet Help Desk.
